# A hybrid technique for measurement of intra/extracellular proteins

**DOI:** 10.1371/journal.pone.0282948

**Published:** 2023-05-04

**Authors:** Haiam Edabashi, Radwa Elghadafi, Nischwethaa Rajkanth, Janvi Saurabh Mody, Weiyuan Ma, Serge Dibart, Xiaoren Tang

**Affiliations:** Department of Periodontology, Henry M. Goldman School of Dental Medicine, Boston University, Boston, MA, United States of America; Purdue University, UNITED STATES

## Abstract

ELISA or Western blot is known as a basic technique to be used for measurement of intracellular proteins, but in some cases, they cannot overcome problems such as normalization between samples or extraneous costs for required commercial kits. In order to address this problem, we developed a rapid and effective method (a hybrid of Western blot and ELISA). We use this new hybrid method to detect and normalize trace protein changes in gene expression intracellularly at a lower cost.

## Introduction

It is known that ELISA (enzyme-linked immunosorbent assay) is an assay technique used to measure proteins, which is commercially available in various different kits [1]. Utilization of the correct corresponding ELISA kit is a more convenient and fast method to measure/determine trace amounts of proteins without using many facilities and equipment. However, we can occasionally encounter difficulties such as the normalization of the relative protein levels among multiple test groups. Additionally, the cost of the commercial ELISA kit is very expensive. We know another method such as western blot that can also be used to measure protein level of gene expression [2]. It seems that western blot can solve these problems addressed above such as, normalization and cost. However, there are another problem with using western blot. Unlike ELISA that can be completed in a few hours, usually takes one or more days to complete the western blot procedure. Western blot detects the amount of protein with the image signal, which can only be estimated visually and often impossible to be accurately quantified. Also, western blot cannot be accomplished without some large facilities and equipment such as, running protein system, transferring protein system, film developer, and image system. Thus, we were interested in measurement of proteins at varying quantities intracellularly by a hybrid method that can combine the main features of ELISA and western blot while avoiding their main weaknesses.

In this study, a rapid, effective and hybrid method was developed by our group, which can be used to detect and normalize much smaller protein changes in gene expression intracellularly at a low cost without using facilities that is required for western blot.

## Material and methods

Mouse RAW264.7 macrophage-like cells (Cat# TIB-71™, ATCC®) or human THP-1 monocyte-like cells (Cat# TIB-202™, ATCC®) were grown in RPMI medium 1640 (Cat#: 11875–093, Life Technologies, NY) with 10% FBS (Fetal Bovine Serum, Sigma) at 37°C in a 5% CO_2_ atmosphere.

**ELISA analysis**: 1x10^5^ mouse RAW cells were treated with 0.1μg/ml *E*.*coli* LPS for 0–36 hours. The treated media from each test group were collected at different time points (0, 6, 12, 24, or 36 hours) according to their respective experiments and tested with ELISA kit (Cat#: ab208348 for mouse TNF-α, Abcam) following manufacturer’s instruction. Its immuno-reactivity was quantified by using a microplate reader (Victor3 TM 1420, BioSurplus) with its respective program (Wallac 1420 Manager, Perkin Elmer Life and Analytical.com) and graphed. **Western blot analysis:** The protein from the same treated cells as in ELISA were purified and detected with antibodies against TNFα (sc-1351, Santa Cruz) or Actin (sc-8432, Santa Cruz) as control. Intensity of protein band was measured, labeled and analyzed using VersaDoc Imaging System model 4000MP with Quantity One Quantitation Software version 4.6.3 (Bio-Rad). **Hybrid method analysis for intracellular protein:** The treated cells were collected and lysed with Lysis Reagent (Cat.# E1500, Promega). ≤2μg/100 μl cell lysate for each sample was regulated and loaded into a well of 96-well microplate (Cat #: 5665–5161, USA Scientific Inc) and fixed with 7% Formaldehyde solution for 20–40 mins. The fixing solution was removed and washed three times with 100 μl 1x Wash Buffer (1x Tris-Buffered Saline+0.1% Tween® 20 Detergent, TBST) for five minutes per wash cycle. 100 μl of blocking buffer was added (1xTBS+ 5% w/v non-fat dry milk) and incubated for 1 hour at room temperature. The blocking buffer with 100 μl was removed with 1x wash buffer and washed three times. 0.1 μg/ml of primary antibody, TNF-α (sc-1351, Santa Cruz), IL-1α (sc-1254, Santa Cruz), IL-10 (sc-1783, Santa Cruz), Caspase 3 (sc-7148, Santa Cruz), Caspase 9 (sc-7885, Santa Cruz), p21 (sc-817, Santa Cruz), Bax (sc-6236, Santa Cruz), c-jun (#9165, Cell Signaling), MyD88 (sc-8196, Santa Cruz), CXCL1 (Cat#:PA5-28822), or Actin (sc-8432 Santa Cruz) as control was individually added in each well of 96-well microplate with 100 μl TBS plus 5% w/v nonfat dry milk, then incubated for 1 hour at room temperature. The primary antibody was removed with 100 μl 1x wash buffer and washed three times, each time for five minutes by shaking at 125-150rpm on the shaker (Incu-Shaker Mini, Benchmark). 0.01μg of the corresponding secondary antibody with 100 μl TBS+5% w/v nonfat dry milk was added and incubated for 30 minutes at room temperature. The secondary antibody was removed with 100 μl 1x wash buffer and washed three times, each time for five minutes by shaking at 125-150rpm on the shaker. 100 μl of Ready-to-Use Substrate (Lot #: 193332, LSBio) was added to each well and incubated for about 30 minutes at room temperature in the dark. Finally, added 100 μl of Stop Solution (Lot#: 188996, LSBio) to each well. The concentration of TNF-α, Actin or others from each well were further identified and analyzed by microplate reader (Victor3 TM 1420, BioSurplus) with its respective program (Wallac 1420 Manager, Perkin Elmer Life and Analytical, com) and graphed. **Hybrid method analysis for extracellular protein**: The media from treated cells were collected and fixed with 7% Formaldehyde solution for 20–40 mins. The fixing solution was removed and washed three times with 100 μl 1x Wash Buffer (1x Tris-Buffered Saline+0.1% Tween® 20 Detergent, TBST) per wash cycle. 0.1 μg/ml of primary antibody, TNF-α (sc-1351, Santa Cruz), IL-1β (sc-515598, Santa Cruz), or Actin (sc-8432 Santa Cruz) as control was individually added in each well of 96-well microplate with 100 μl TBS plus 5% w/v nonfat dry milk, then incubated for 1 hour at room temperature. The primary antibody was removed with 100 μl 1x wash buffer and washed three times. 1:2,000 ratio diluted corresponding HRP conjugated secondary antibody (W4021, Promega) with 100 μl TBS+5% w/v nonfat dry milk was added and incubated for 30 minutes at room temperature. The secondary antibody was removed with 100 μl 1x wash buffer and washed three times. 100 μl of Ready-to-Use Substrate (Lot #: 193332, LSBio) was added to each well and incubated for about 30 minutes at room temperature in the dark. Finally, added 100 μl of Stop Solution (Lot#: 188996, LSBio) to each well. The further identified protein was analyzed by microplate reader (Victor3 TM 1420, BioSurplus) with its respective program (Wallac 1420 Manager, Perkin Elmer Life and Analytical, com) and graphed. **Statistical analysis:** All experiments were performed in triplicate and statistical analyses were conducted with the SAS software package. All data was normally distributed. For multiple mean comparisons, we conducted analysis of variance (ANOVA), while we used the Student’s t-test for single mean comparison. For time-course study, we used a two-way repeated measure ANOVA. P values less than 0.05 was considered significant.

## Results

### Fixation of cell lysate by Formaldehyde

**Fig 1:**
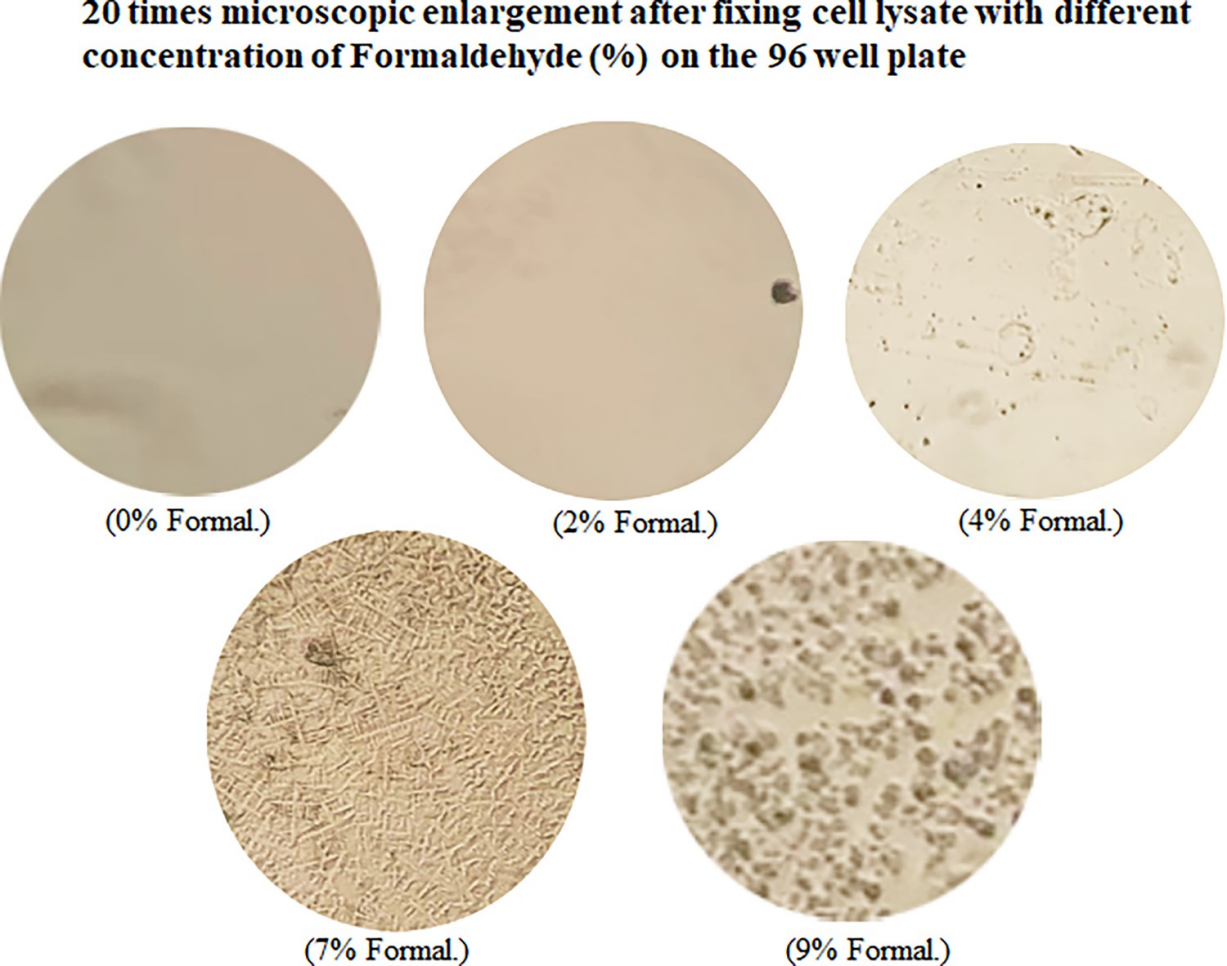
Microscopic observation of the fixed shape of the cell lysate protein after Formaldehyde treatment. The pre-cultured RAW cells were lysed with lysis buffer. 5 μg/100 μl cell lysate plus different concentration (0%, 2%, 4%, 7%, or 9%) of Formaldehyde was added in wells of 96-well microplate for 30 m. The fixed cell lysate in each well was washed with the washing buffer. Photomicrograph of Formaldehyde-fixed cell lysate was taken at ×20 magnification.

It is known that cell lysate/protein can be fixed on the commercial 96-well microplate with 4% of Formaldehyde solution for immuno-histological analysis or ELISA analysis [3] but the question still remains whether 4% Formaldehyde is an ideal concentration for cell lysate fixation. Thus, we treated cell lysate with different concentration of Formaldehyde solution for 30 min to test the resulting effect on cell lysate fixation. As shown in Fig 1, 2% Formaldehyde solution-treated cell lysate had no effect of fixation, and 4% Formaldehyde solution-treated cell lysate was less than 20% effective. However, the fixation effect of 7% Formaldehyde solution on treated cell lysate was notably higher, approximately 85% or more. The fixation effect of 9% Formaldehyde solution on treated cell lysate was also high (lower than that of 7% Formaldehyde solution) but the fixed form appeared abnormal (unknown reason). It suggests that 7% Formaldehyde solution is a suitable concentration for cell lysate fixation.

### Detection of protein without false positives

**Fig 2:**
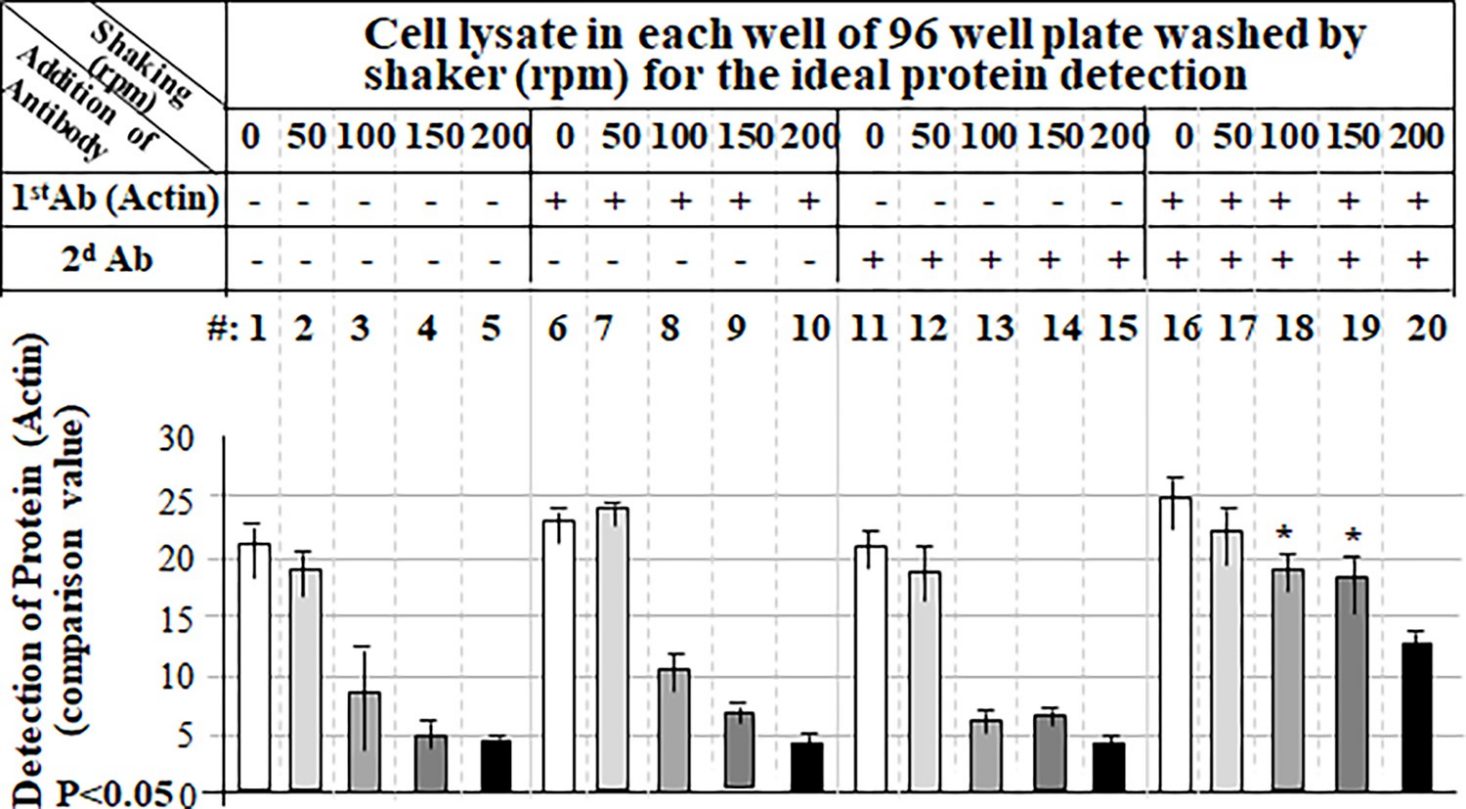
Specific detection of protein by our hybrid method without false positive. 2 μg/100 μl cell lysate fixed with 7% Formaldehyde in a well of 96-well microplate was untreated as control (No.1-5) or treated with Primary (1st) antibody alone (No. 6–10) or secondary (2d) antibody alone (No. 11–15) or both 1st and 2d antibodies (No. 16–20). After treatment with each antibody, each test cell lysate in the well was added with the washing buffer and washed by shaking at different rotation per minute, 0 rpm No. 1, 6, 11, or 16), 50 rpm (No. 2, 7, 12, or 17), 100 rpm (No. 3, 8, 13, or 18), 150 rpm (No. 4, 9, 14, or 19), or 200 rpm (No. 5, 10, 15, or 20). The concentration of Actin on each well was respectively analyzed by a microplate reader (Victor3 TM 1420, BioSurplus) with its respective program Wallac 1420 Manager, Perkin Elmer Life and Analytical.com. The expression value of Actin on each well was further calculated compared with the control and graphed with their rate.

**Fig 3:**
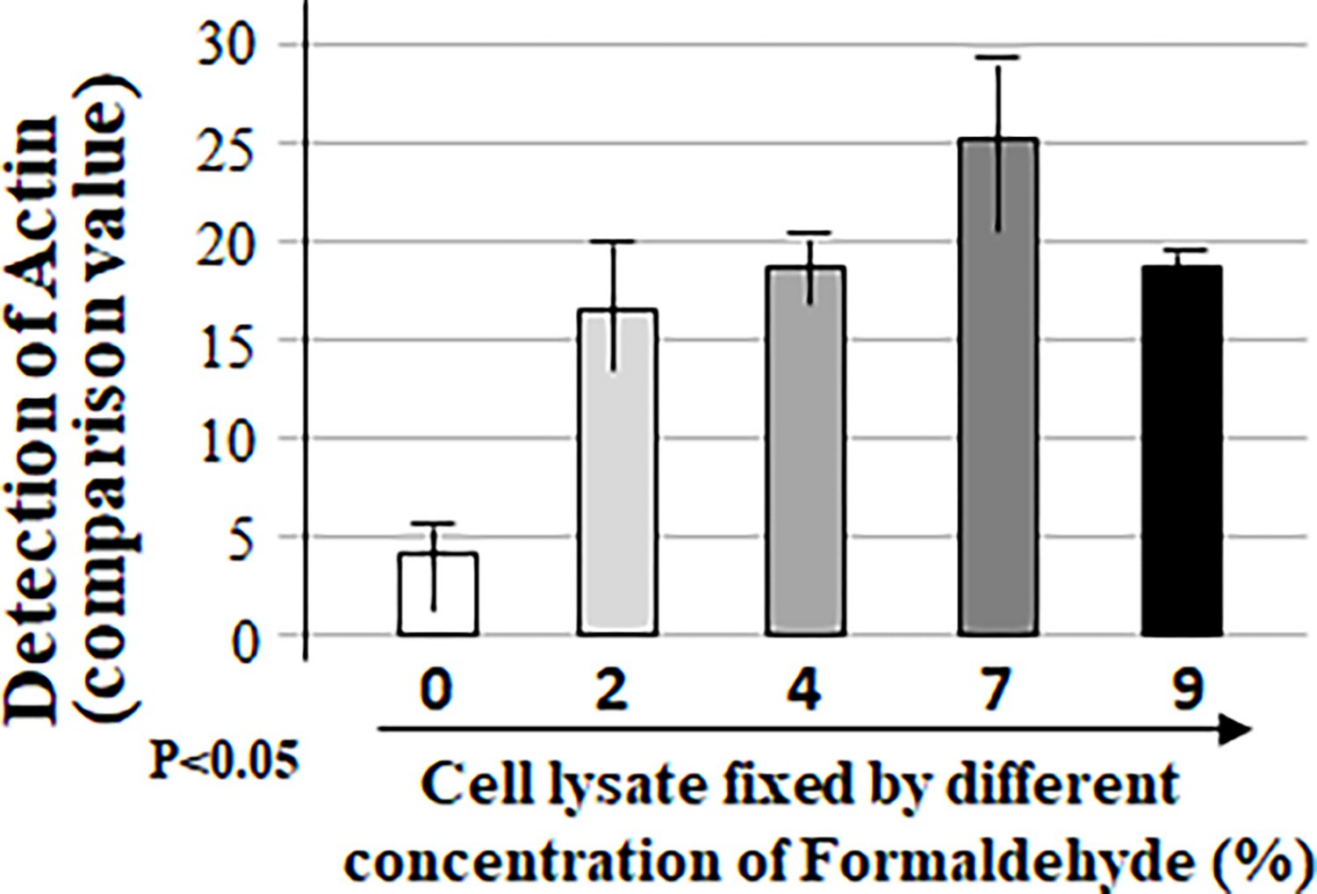
Examination of the favorable condition 1 for this hybrid method. Five groups of lysate samples (5μg of cell lysate/group) were added into the wells of 96-well microplate, fixed with different concentrations of formaldehyde solution (0%, 2%, 4%, 7% or 9%) for 30 minutes, treated with Actin (primary antibody) and corresponding antibody as described (Fig 2, No.19), then the protein from each test group was detected by this hybrid method.

**Fig 4:**
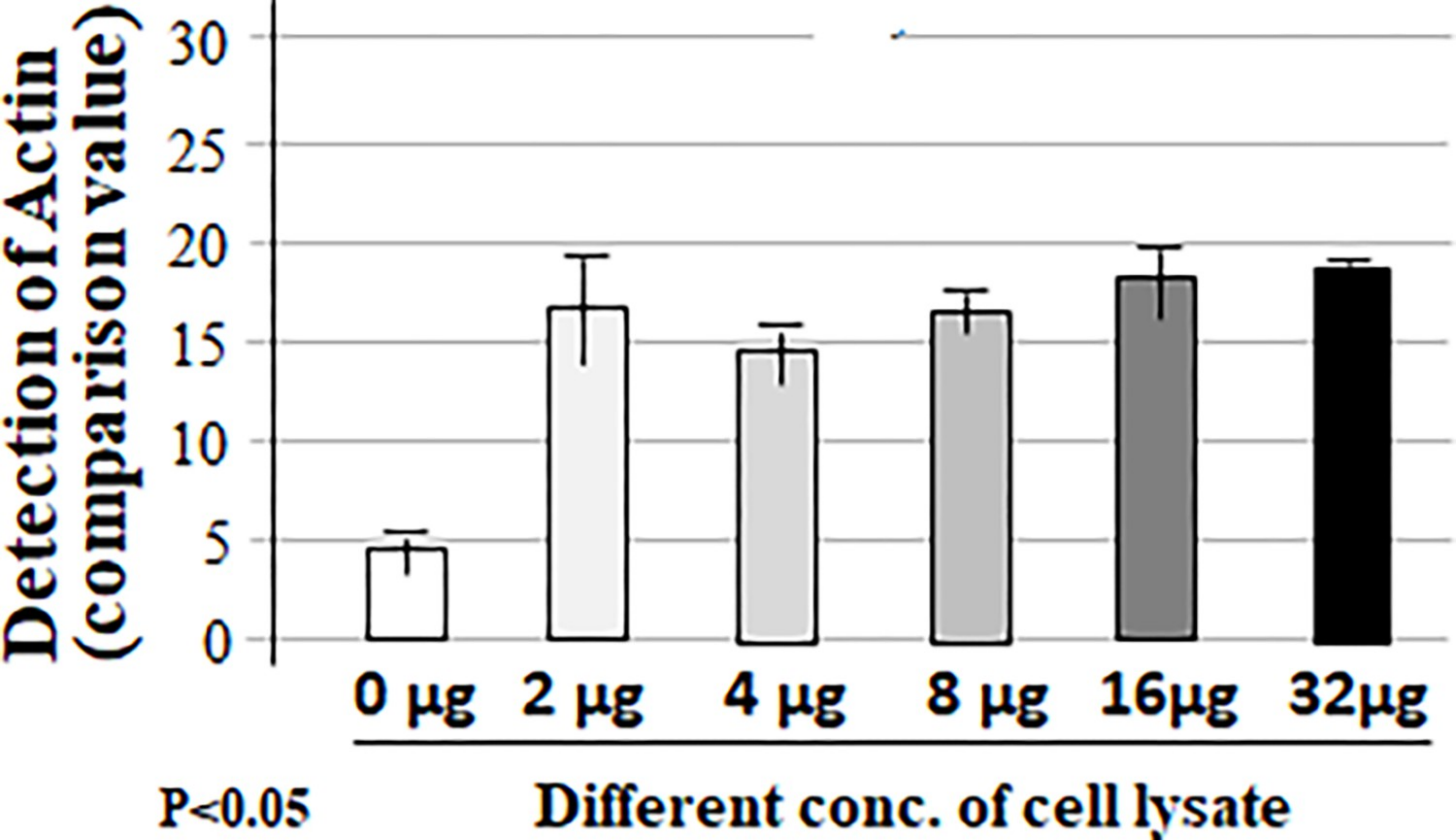
Confirmation of the favorable condition 2 for this hybrid method. The different concentration (0, 2, 4, 8, 16, or 32 μg) of cell lysate samples were respectively added into a well of 96-well microplate and fixed with 7% Formaldehyde solution for 30 min, treated with Actin (primary antibody) and corresponding antibody, then analyzed by this hybrid method.

**Fig 5:**
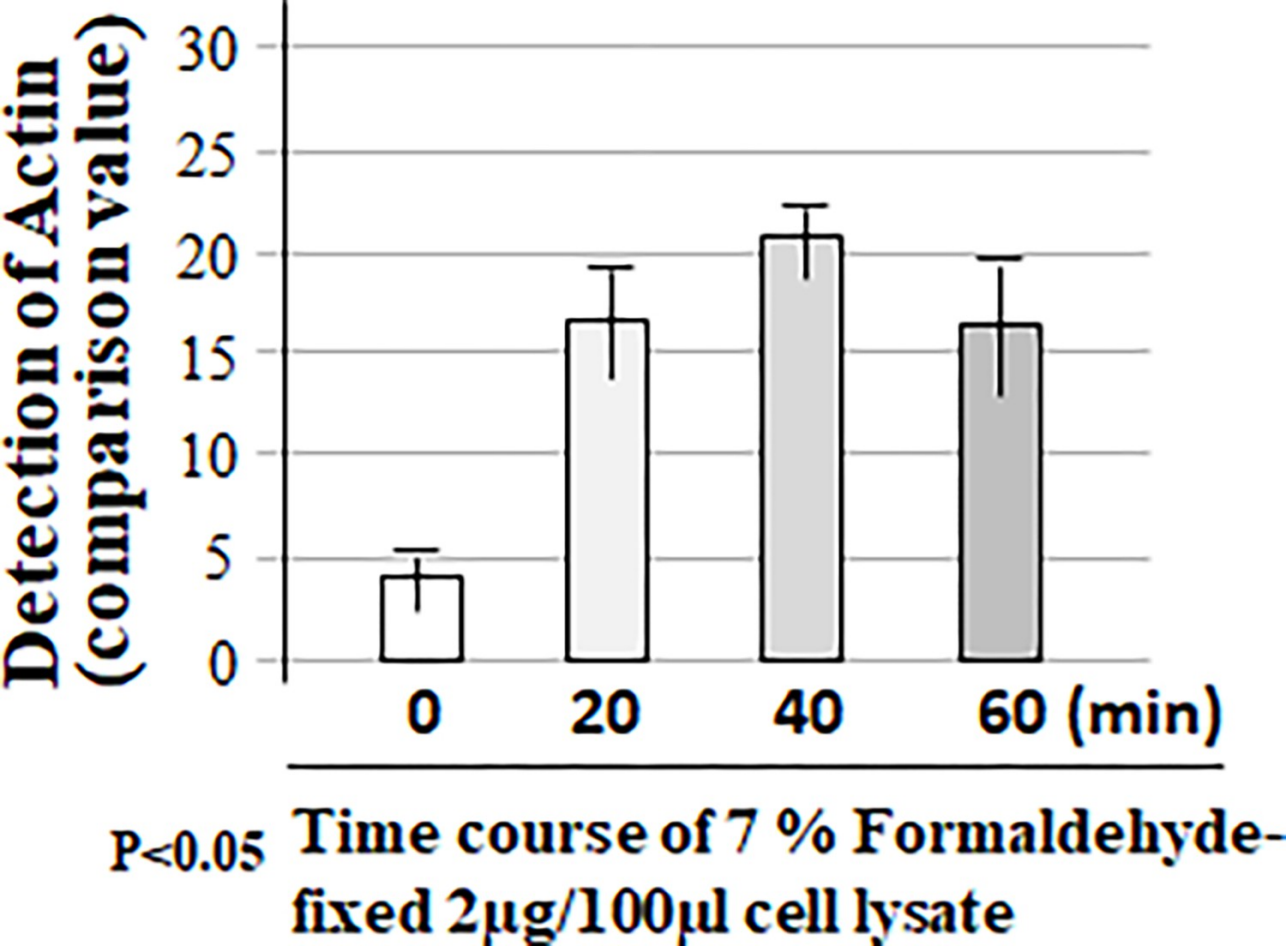
Confirmation of the favorable condition 3 for this hybrid method. Four groups of cell lysate samples (2μg of cell lysate/group) were respectively added into a well of 96-well microplate and fixed with 7% Formaldehyde solution for 0, 20, 40, or 60 min. After treatment of with Actin (primary antibody) plus corresponding antibody, the protein was detected by this hybrid method.

To check whether the cell lysate fixed in a well of the regular 96-well microplate (not commercial ELISA plate) can be detected without non-specificity or false positives with an appropriate washing condition, we conducted a more rigorous experiment following the experimental design shown in the Table above (Fig 2). Cell lysate in each well of 96-well microplate was untreated or treated with only primary antibody (Actin) or the corresponding second antibody as negative control or treated with both 1st and 2nd antibodies as test group. Samples in 96-well microplate were also spun down by the shaker at varying speeds between 0–200 rpm. As shown in Fig 2, when the shaking speed was 50 rpm or below, the value of protein detection with adding only Actin antibody (No. 6 & 7, 11 & 12) and/or both Actin and proper corresponding 2nd antibodies (No. 16 & 17) was relatively high and almost the same to the value without antibodies (No. 1 & 2). However, when the shaking speed was up to 100 rpm or more, the cell lysate treated without antibody (No. 3 & 4) or with Actin alone (No. 8 & 9) or 2nd antibody alone (No. 13 & 14) was undetectable compared to the value of protein detection with adding both Actin and 2nd antibodies as test group (No. 18 & 19). It suggests that the 7% Formaldehyde solution fixed cell lysate in the regular well of 96-well microplate with a more stringent washing condition (shaking at 100-150rpm) can be specifically detectable by antibodies without a false positive. This new method with a combination of western and ELISA proved to be very effective for protein measurement. We further tested the conditions for this hybrid method. As shown in Figs 3–5, a more favorable condition of Actin protein detection was confirmed while 2 μg of cell lysate (Fig 3) in a well of 96-well microplate was fixed by 7% Formaldehyde solution (Fig 4) with the same washing condition (shaking at 125 rpm) for 40 min (Fig 5).

### Comparison among ELISA, Western and this hybrid method

**Fig 6:**
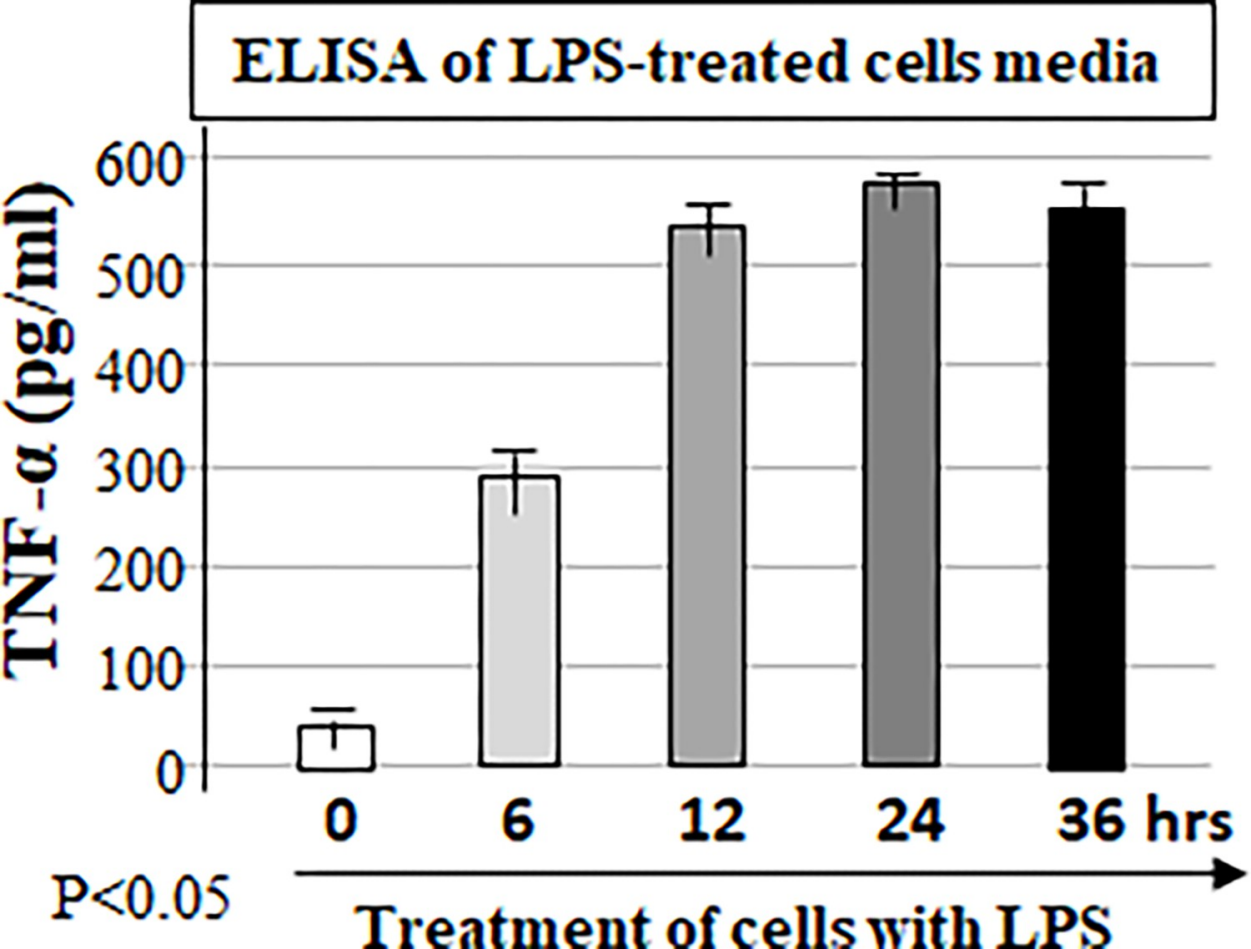
ELISA analysis of LPS-induced TNF-α. The pre-cultured 1x10^5^ RAW cells were treated with 0.1 μg LPS for 1 hour and washed twice with PBS, then continued to culture with fresh media for 0, 6, 12, 24, or 36 hours. The media were collected from each test group at different time point and used for detection of mouse TNF-α concentration by ELISA.

**Fig 7:**
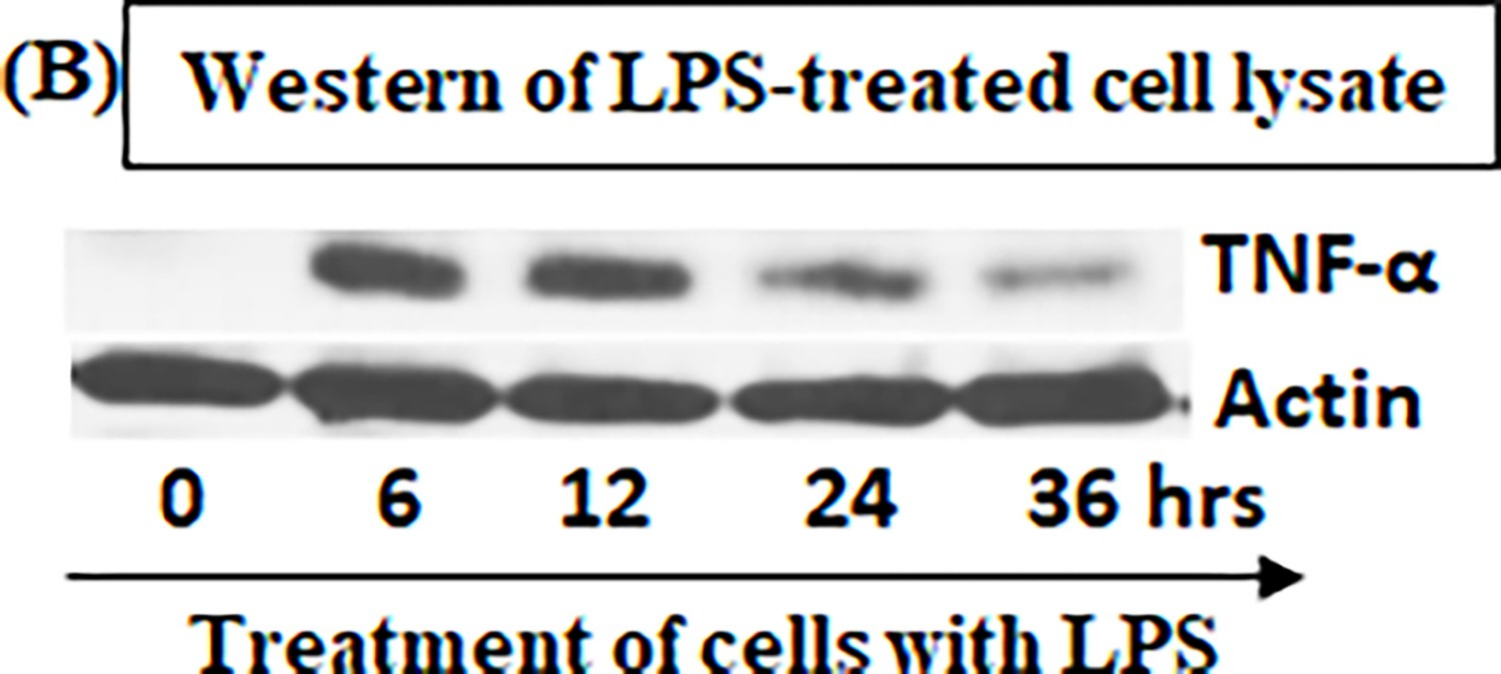
Western blot analysis of TNF-α or Actin in response to LPS. The cell lysate was collected from each test group at the condition with different time point as described (Fig 6) and used for detection of mouse TNF-α or Actin by Western blot.

**Fig 8:**
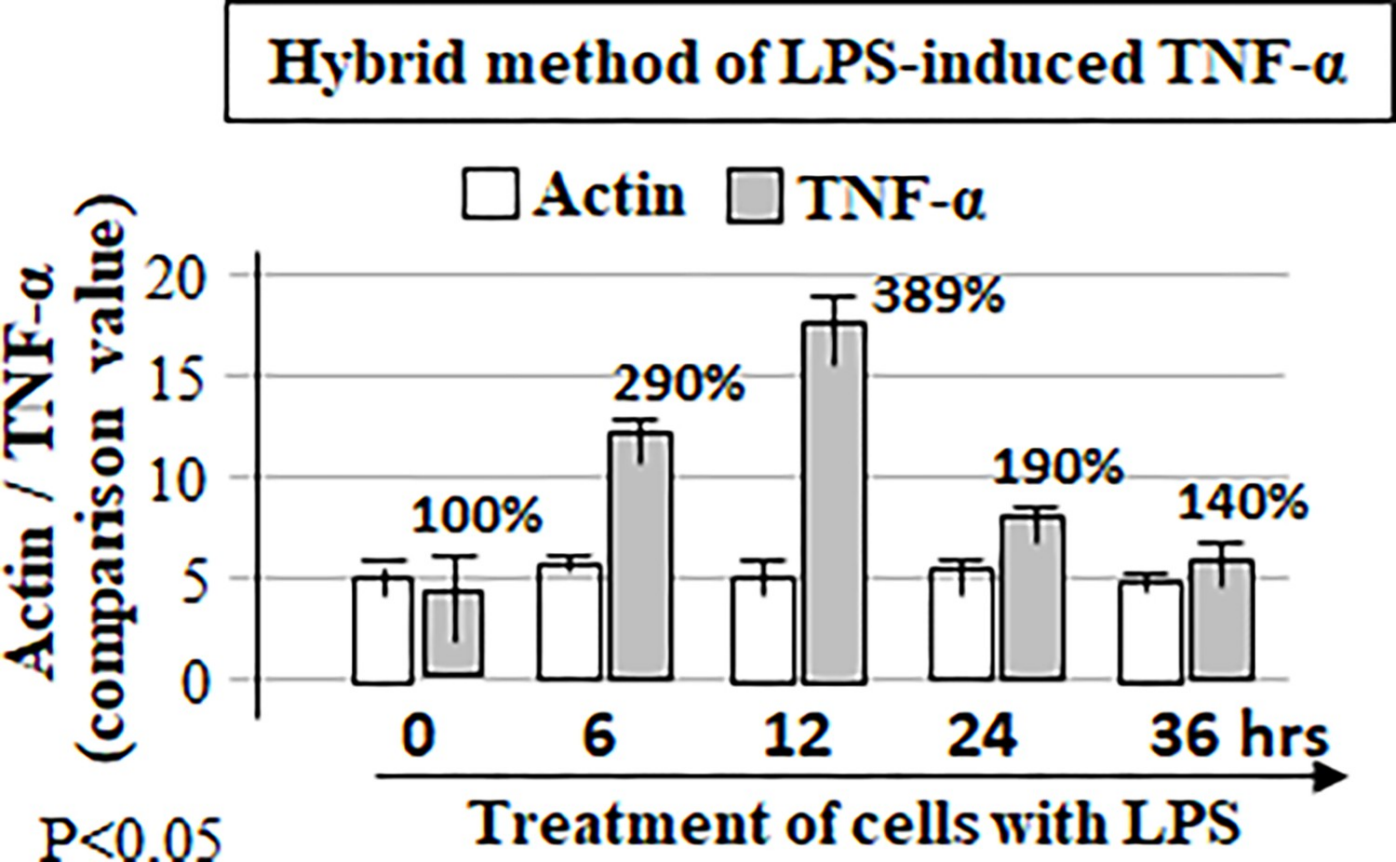
Analysis of LPS-induced TNF-α or Actin by this hybrid method. The cell lysate was collected from each test group at the condition with different time point as described (Fig 6) and used for detection of mouse TNF-α or Actin by this hybrid method.

**Fig 9:**
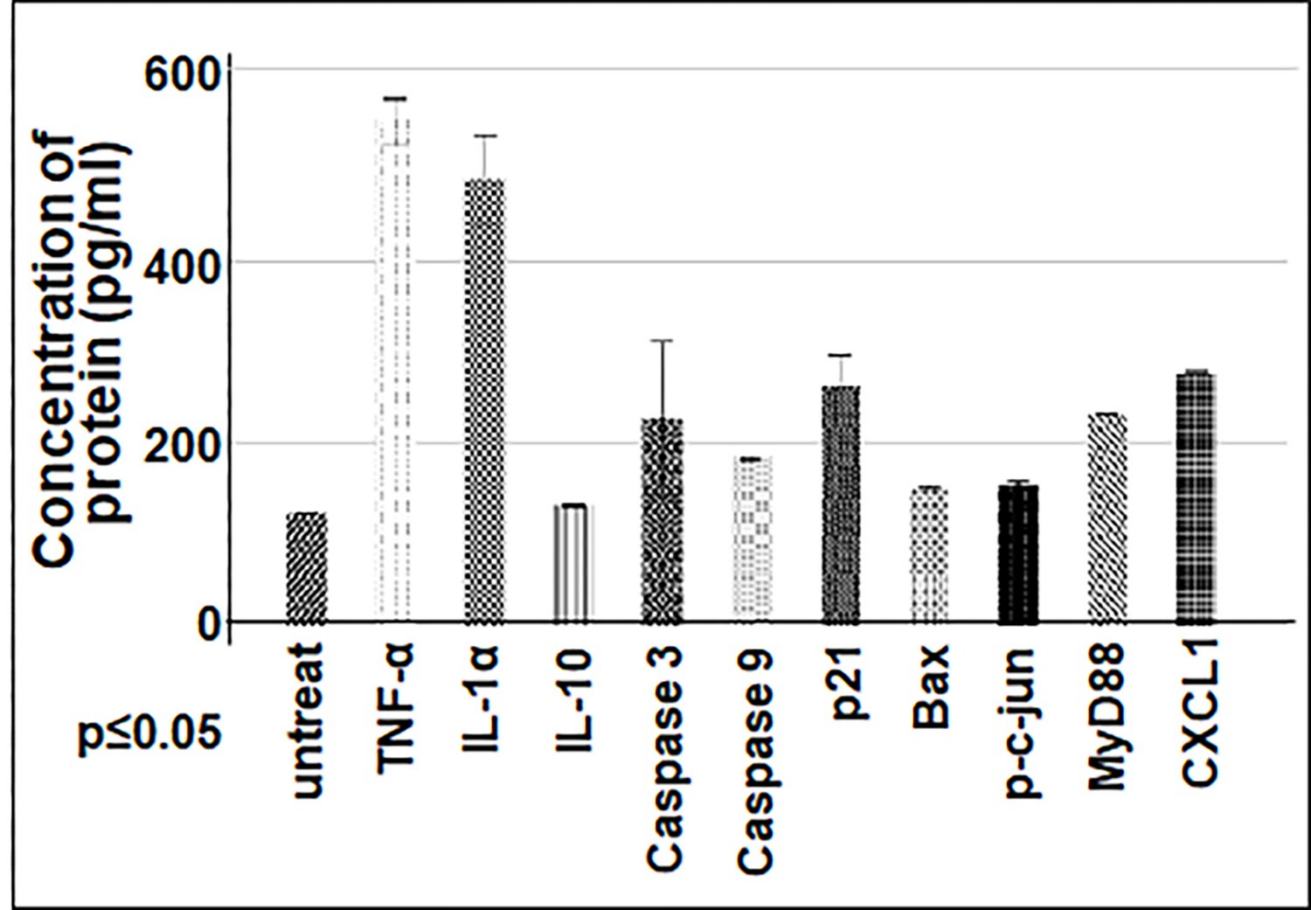
Protein assay by this hybrid method. 2 μg/100 μl cell lysate fixed with 7% Formaldehyde into each well of 96-well microplate was untreated as control or treated with Primary antibody (TNF-α, IL-1α, IL-10, Caspase 3, Caspase 9, p21, Bax, c-jun, MyD88, or CXCL1 for 1 hour, and added with its corresponding secondary antibody for 30 min, then analyzed by this hybrid meth and graphed with their rate.

In order to compare the difference between the hybrid method and western/ELISA, the pre-cultured RAW cells were treated with *E*.*coli* LPS for 0, 6, 12, 24, or 36 hours. ELISA analyzed the media collected from each test group at different time points with antibodies against TNF-α (Fig 6). The cell lysate from each test group at different time point was detected with antibodies against TNF-α or Actin as control by western blot (Fig 7) or by this hybrid method (Fig 8). With ELISA analysis, the treatment of cells with LPS secreted TNF-α production increased by 6 fold at 6 hours, 14 fold at 24 hours or 13.5 fold at 36 hours when compared to the control (cells treated for 0 hour, Fig 6). It is clear from the results that the LPS-induced TNF-α secretion had continued to increase in 6 hours, peaked at 24 hours, and remained at the same secretory level from 24–36 hours. However, it is not like the extracellular TNF-α production detected by ELISA, the results from western blot showed that LPS-induced intracellular TNF-α production progressively increased during 6 to 12 hours of treatment, but the protein level of TNF-α decreased to the baseline level after 24 hours when compared to the control (cells treated for 0 hour, Fig 7). A similar phenomenon was observed from the western blot with the hybrid method (Fig 8). It suggests that the result obtained by ELISA does not reflect the true TNF-α expression at the different time point in response to LPS, but the utilization of Western blot or our hybrid method may resolve this shortcoming. To test the practicability of this hybrid method we also tested the effect of LPS in THP-1 cells on the amount of the intracellular protein and showed good results (Fig 9).

### Analysis of extracellular protein in treated media

**Fig 10:**
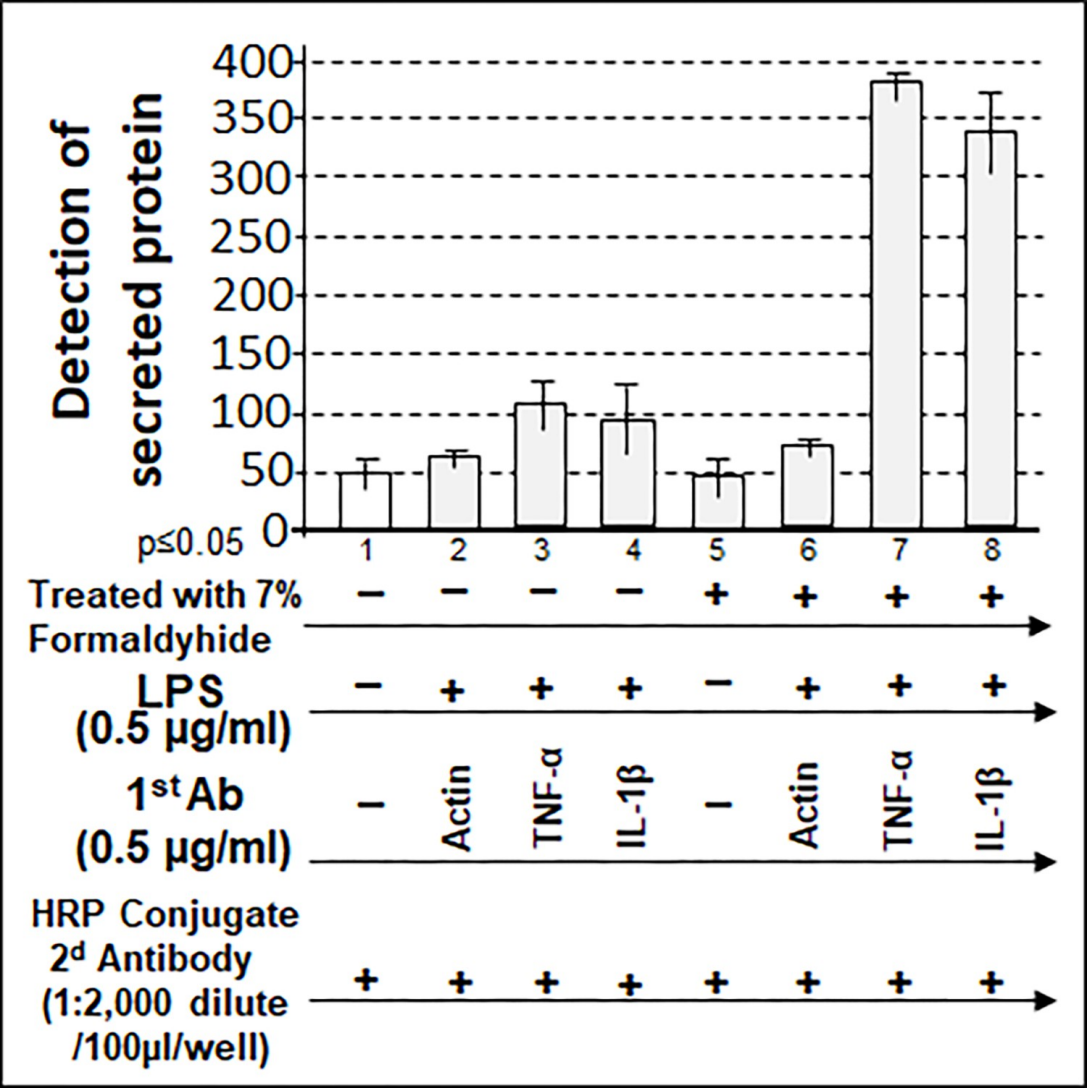
Detection of extracellular protein by this hybrid method. 100 μl of 7% Formaldehyde-fixed media was untreated as control or treated with Primary antibody (TNF-α, IL-1β or Actin for 1 hour, washed and added with its corresponding HRP conjugated secondary antibody for 30 min. The result was analyzed and graphed with their rate.

To check whether this hybrid method can be used to detect the extracellular protein, the unfixed media (No. 1–4) or 7% Formaldehyde-fixed media (No.5-8) was untreated or treated with LPS, then detected by the primary Ab and HRP conjugated secondary Ab. As shown in Fig 10, LPS-induced TNF-α/IL-1β as secreted extracellular protein in 7% Formaldehyde-fixed media (N0. 7 & 8) was significantly detected compared to the untreated media (No. 3 & 4). Additionally, Actin was not able to be detected in either untreated with 7% Formaldehyde (No. 2) or treated media (No.6). It suggests that there should be no a detectable Actin in the culture medium since Actin is an intracellular protein.

## Discussion

A hybrid of Western blot with ELISA has been recently reported to quantify the accuracy of protein in 96-well microplate for some experiments such as In-situ Protein Detection or In-cell Western (ICW) [4, 5]. However, this hybrid technique has only been applied to cells cultured on 96-well microplates and its reproducibility of the data can be greatly affected by intermediate steps or uncontrolled factors, such as protein sample preparation, protein fixation, normalization, or false positives.

The following experimental results in this paper show that usage of this hybrid method is capable of effectively resolving the above-mentioned possible problems. ELISA analysis in Fig 6 shows that the amount of TNF-α protein secreted by LPS-treated cells remained at a high expression level from 24 hours to 36 hours. However, the results obtained by the Western blot (Fig 7) showed that LPS-induced intracellular TNF-α no longer had induction during the period of 24 hours to 36 hours, and its gene expression had dropped to base line expression level. TNF-α expression protein obtained in the above two different experiments had completely different results, which makes it indeterminate whether treatment with LPS still induces TNF-α production after 24 hours. When using our hybrid method to measure TNF-α induction under the same conditions, the result is not only similar to the western blot results, but also the amount of protein change is precisely calculated. It suggests that the short interval time of 1–2 hours for treatment of cells with LPS can only induce TNF-α gene expression within 24 hours and this induction no longer occurs after TNF-α gene reverts to a non-expression level.

With usage of an ELISA commercial kits, due to cost reasons, only one protein (such as TNF-α) is usually measured (see Fig 6), that is, two or more proteins (such as TNF-α plus Actin as control) are not detected at the same time by using one commercial kit. Therefore, the results from ELISA analysis sometimes are difficult to be further normalized. Fortunately, this shortcoming appears to be resolved with our hybrid method (see Fig 8). Furthermore, using this hybrid method, we have simultaneously measured 11 proteins of cells in one well of 96-well microplate within 24 hour time interval (Fig 9).

In addition, although some research groups such as Chowdhury et al. [4], Mukherjee et al. [5], and Huebinger et al. [6], have reported the method such as ICW for detection of protein in 96-well microplate, they did not mention how to overcome false positives in the experiment. In this paper, the problem of false positives has been overcome by implementing a strict washing condition. Besides RAW cells, we also used our hybrid method to detect the protein concentration for different cell line cells such as U2OS, H1299, 293T, or A549, and obtained favorable results (data not shown). It suggests that this method is indeed suitable for protein detection in various types of cells.

Overall, usage of our hybrid method can help us to detect and normalize intracellular proteins rapidly, efficiently, and effectively at a lower cost.

## Supporting information

S1 Raw image (PDF)
