# Supplementary figures and images for: A hybrid technique for measurement of intra/extracellular proteins
Source: PLoS One. 2023 May 4;18(5):e0282948. doi: 10.1371/journal.pone.0282948 (PMC10159171; doi:10.1371/journal.pone.0282948)

Original figure 7 by Western blot

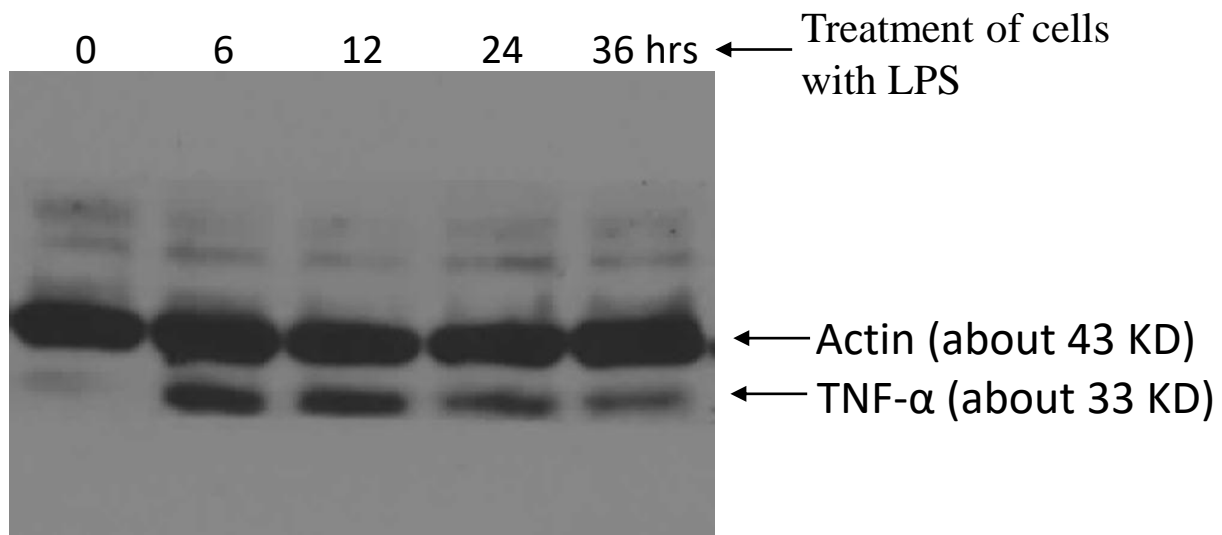

Supplement: S1 Raw image — (PDF) [file pone.0282948.s001.pdf]
